# Second-Order Photoinduced Reflectivity for Retrieval of the Dynamics in Plasmonic Nanostructures

**DOI:** 10.1021/acs.nanolett.2c01478

**Published:** 2022-07-22

**Authors:** Dror Hershkovitz, Uri Arieli, Sudarson Sekhar Sinha, Ori Cheshnovsky, Haim Suchowski

**Affiliations:** ^†^Raymond and Beverly Sackler Faculty of Exact Sciences, School of Chemistry, ^‡^Center for Light−Matter Interaction, ^§^Raymond and Beverly Sackler Faculty of Exact Sciences, School of Physics & AstronomyTel Aviv University, Tel Aviv 6997801, Israel

**Keywords:** plasmonics, metallic nanowires, ultrafast dynamics, pump probe, two-temperature model

## Abstract

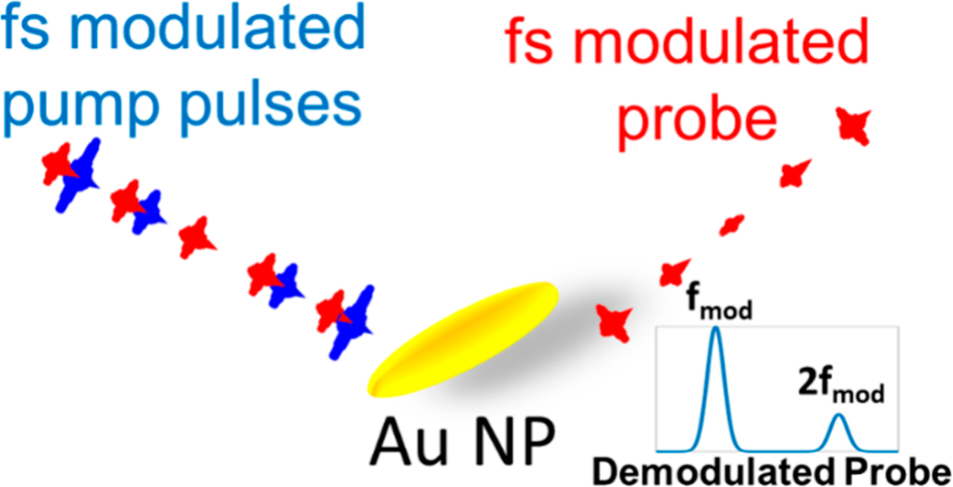

Measuring the change in reflectivity (Δ*R*) using the traditional pump–probe approach can monitor photoinduced ultrafast dynamics in matter, yet relating these dynamic to physical processes for complex systems is not unique. By applying a simple modification to the classical pump–probe technique, we simultaneously measure both the first and second order of Δ*R*. These additional data impose new constraints on the interpretation of the underlying ultrafast dynamics. In the first application of the approach, we probe the dynamics induced by a pump laser on the local-surface plasmon resonance (LSPR) in gold nanoantennas. Measurements of Δ*R* over several picoseconds and a wide range of probe wavelengths around the LSPR peak are followed by data fitting using the two-temperature model. The constraints, imposed by the second-order data, lead us to modify the model and force us to include the contribution of nonthermalized electrons in the early stages of the dynamics.

Ultrafast dynamics in matter, such as photoinduced collective carrier dynamics, femtochemistry, and light-harvesting photosynthesis, are at the research forefront of light–matter interactions. They provide profound insight into correlated electronic dynamics,^1−3^ nonlinear optical generation,^4−6^ and quantum information processing^7,8^ that will benefit the exponential demand for energy -harvesting devices as well as extreme ultrafast interconnects and sensing technologies. In recent years, ultrashort pulses with their inherent ultrabroad spectrum allowed us to unravel ultrafast effects in plasmonic nanostructures,^9−12^ spanning time scales from femtoseconds (fs) to picoseconds (ps). The photoinduced temporal evolution of these interactions consists of several stages. First, the pump pulse excites plasmons, followed by the dephasing of collective coherent electronic motion. Then, the nonthermalized charge carriers exchange energy with other electrons and thermalize. After a few picoseconds, a thermalization process of hot electrons and a cold lattice occurs until the lattice equilibrates with the environment.^13,14^ During these stages, matter experiences dramatic changes in the properties that dictate the dynamics, environment permittivity,^15^ localized plasmon frequency,^10^ and collision rate^10^ as well as the nanostructure’s transient geometry changes.^16^ All of these are strongly coupled and influence the optical response of the nanostructures.

Such an ultrafast photoinduced response is often tracked by pump–probe (P&P) techniques combined with lock-in detection. A modulated ultrafast pulse train, the pump, excites the sample of interest and induces some change in its optical properties. The photoinduced dynamics are then monitored with a lock-in amplifier by the probe, a time-delayed unmodulated weak ultrafast pulse train.^17−19^ Changes in reflectivity or transmission of the probe allow the exploration of many of the ultrafast dynamics in matter. Because the transient changes in the material’s properties (such as the localized plasmon frequency and collision rate) are complex and interleaved with each other, it is very challenging to assign the dynamics of matter uniquely just from measuring Δ*R*.

Here we present a novel experimental P&P method that simultaneously measures both the first- and second-order transient photoinduced transient reflectivity Δ*R*. We utilize our method to retrieve the ultrafast dynamics of plasmonic gold-bar nanostructures by measuring the transient reflectivity over a wide range of probe wavelengths. We show that the additional second-order response imposes strong constraints on the standard interpretation of ultrafast dynamics in these nanostructures, challenging the standard analysis based on the first-order transient Δ*R*. To facilitate the new analysis of the experimental data, we have developed an optimization algorithm that simultaneously fits both first- and second-order transient reflectivity over multiple wavelengths and delay times, with a single set of kinetic and dielectric function parameters of the gold nanobars. We show that using the common functional dependence of the physical optical parameters on the extended two-temperature model variables was not adequate to reproduce the experimental results. Thus, we modify these functional dependencies of the physical optical parameters. In particular, we find that in order to model the experimental results faithfully, we had to include the contribution of nonthermalized electrons to the dielectric response of the system.

Our experimental approach (Figure 1a) extends the standard P&P setup to enable the measurement of the higher-order differential reflectivity, Δ*R*. The key element in this pump–probe technique is the ability to modulate the pump pulse as a very pure harmonic function at a frequency *f*_m_ to ensure that the high harmonics detected in the Δ*R* signal are due to the nonlinear response of the photoinduced probe’s reflectivity. This target is achieved by driving an acousto-optic modulator (AOM) by an arbitrary waveform generator optimized to generate a pure sine intensity modulation of the pump beam.^20^ We use a multidemodulator lock-in amplifier in which we simultaneously measure both the linear (*f*_m_ modulated response) and nonlinear Δ*R* (*nf*_m_, modulated response, *n* = 2, 3,...). This approach to generating a pure sine modulation was developed to monitor nonlinear induced reflectivity for achieving label-free super-resolution microscopy.^21−23^ The current study utilizes it to explore the ultrafast dynamics of electrons in plasmonic nanostructures.

**Figure 1:**
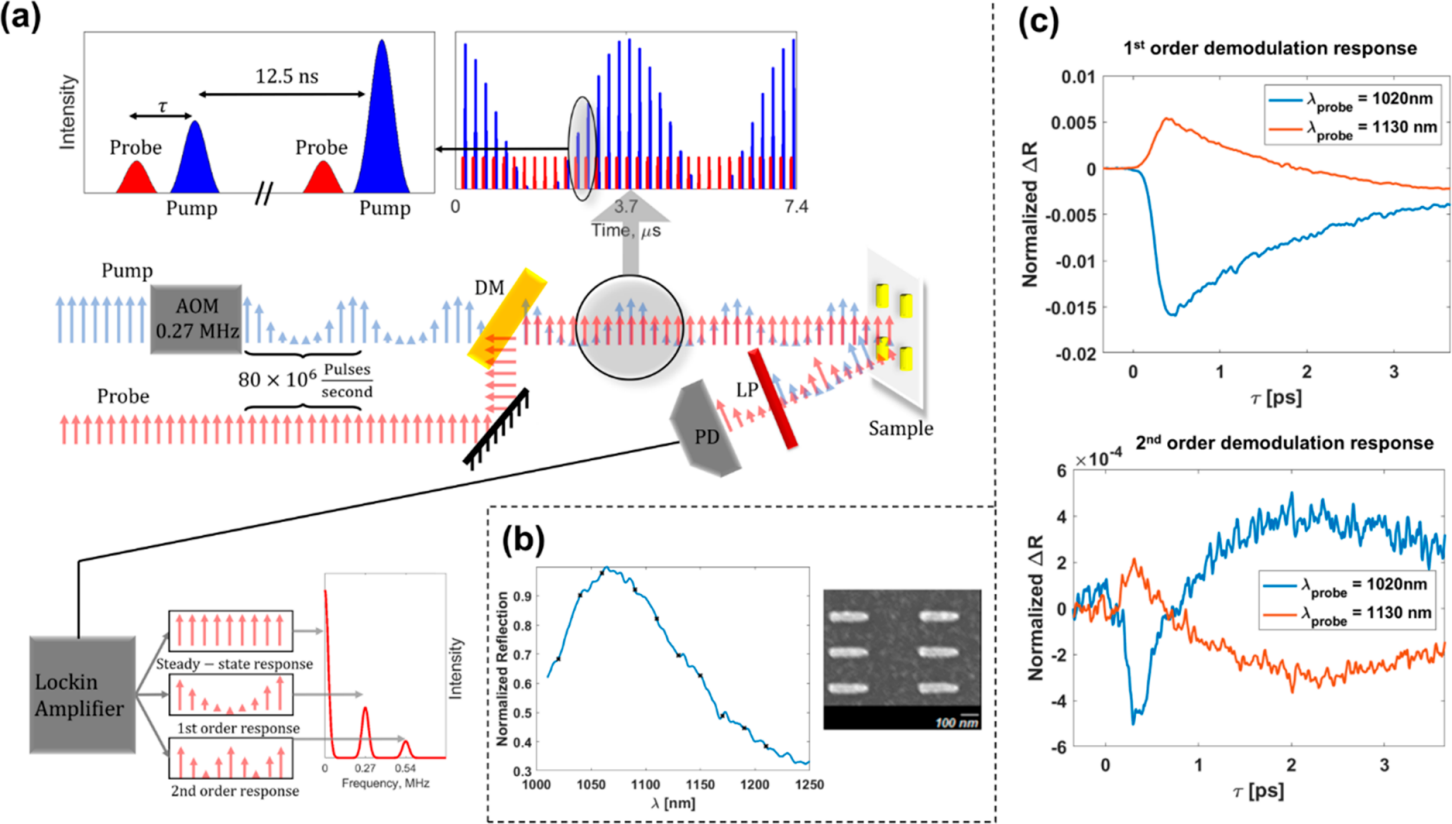
(a) Schematic drawing of the experimental apparatus: high-repetition rate pump pulses (80 MHz) are directed through an AOM coupled to an arbitrary waveform generator to produce a pure harmonic intensity modulation at 0.27 MHz frequency. The pump and an unmodulated IR probe beam are combined using a dichroic mirror and directed onto the sample of the gold nanobar array. The pump reflectance is discarded using a low-pass optical filter, and the changes in the probe reflectance are measured with a photodiode and fed into a multidemodulator lock-in amplifier. By locking onto the first and second harmonics of the modulation frequency, we can measure the first- and second-order responses of the probe Δ*R*. We repeated this experiment over 10 different wavelengths across the nanobar LSPR (marked as black dots on the nanobar spectrum inset). (b) Characteristics of the plasmonic nanostructures. (Left) Gold nanobar array LSPR spectrum. Black dots, marked on the spectrum, signify the probe wavelength used for which Δ*R* was measured in our experiment. (Right) Closeup of the SEM image on the gold nanobar array sample. (c) Single-wavelength measurement of time-domain Δ*R* as a function of pump/probe delay showing the first- and second-order responses (top and bottom, respectively). The blue line is for λ_probe_ = 1020 nm < λ_LSPR_, and the orange line is for λ_probe_ = 1130 nm > λ_LSPR_.

We measure the first- and second-order photoinduced Δ*R* from an array of gold nanobars fabricated over an ITO-covered glass substrate characterized by localized surface plasmon resonance (LSPR) at 1080 nm (Figure 1a). The array of gold nanobars (180 × 40 × 40 nm^3^) with 280 and 130 nm pitches in the *x* and *y* directions, respectively, is pumped with a 100 fs, 800 nm pump train at 80 MHz (Mai-Tai Spectra-Physics) and probed by a variable-wavelength train (tunable IR OPO, 100 fs, 1000–1280 nm) at normal incidence with spatial overlap and a controlled time delay. Both beams are polarized along the long axis of the nanobars and extend spatially over ∼20 nanobars. The pump is sine-modulated by the AOM at *f*_m_ = 271 kHz. A photodiode monitors the induced Δ*R* in probe reflection and is fed to a multidemodulator lock-in, locked to both the principal (*f*_m_) and its second harmonic (2*f*_m_), thus measuring both first- and second-order responses of Δ*R* (Figure 1a). We have performed the transient measurements at different probe wavelengths across the LSPR (1020–1200 nm, Figure 1b spectrum inset), with time delay steps of 10 fs over the range of -0.5–10 ps.

Figure 1c depicts an example of the first- and second-order transient Δ*R* responses from the gold nanobar array with a probe wavelength that is lower (blue line) and higher (orange line) than the LSPR’s wavelength, λ_LSPR_. The linear response shows the expected^9^ decrease/rise in reflectivity in the first ∼700 fs after pump excitation, followed by an exponential decay. Note that the second-order nonlinear response exhibits a faster transient (∼300 fs), in which Δ*R* changes signs.

To validate that the second-order response contains nontrivial physical information, we first extracted the real and imaginary parts of the dielectric function using the complete experimental set of Δ*R* (Figure 2(a,b)). First, we fit our data using a simple empirical model in which we attribute the changes in reflectivity to changes in the gold nanobar permittivity, which are linear with the pump intensity,
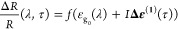
1where  is the gold nanobar permittivity before excitation, *I* is the normalized, unitless pump intensity, and **Δε**^(**1**)^ is a complex fitting parameter for the pump-induced change in ε_g_. We use Mie scattering theory to calculate the dependence of Δ*R*/*R* on ε_g_ (Supporting Information SB).

**Figure 2:**
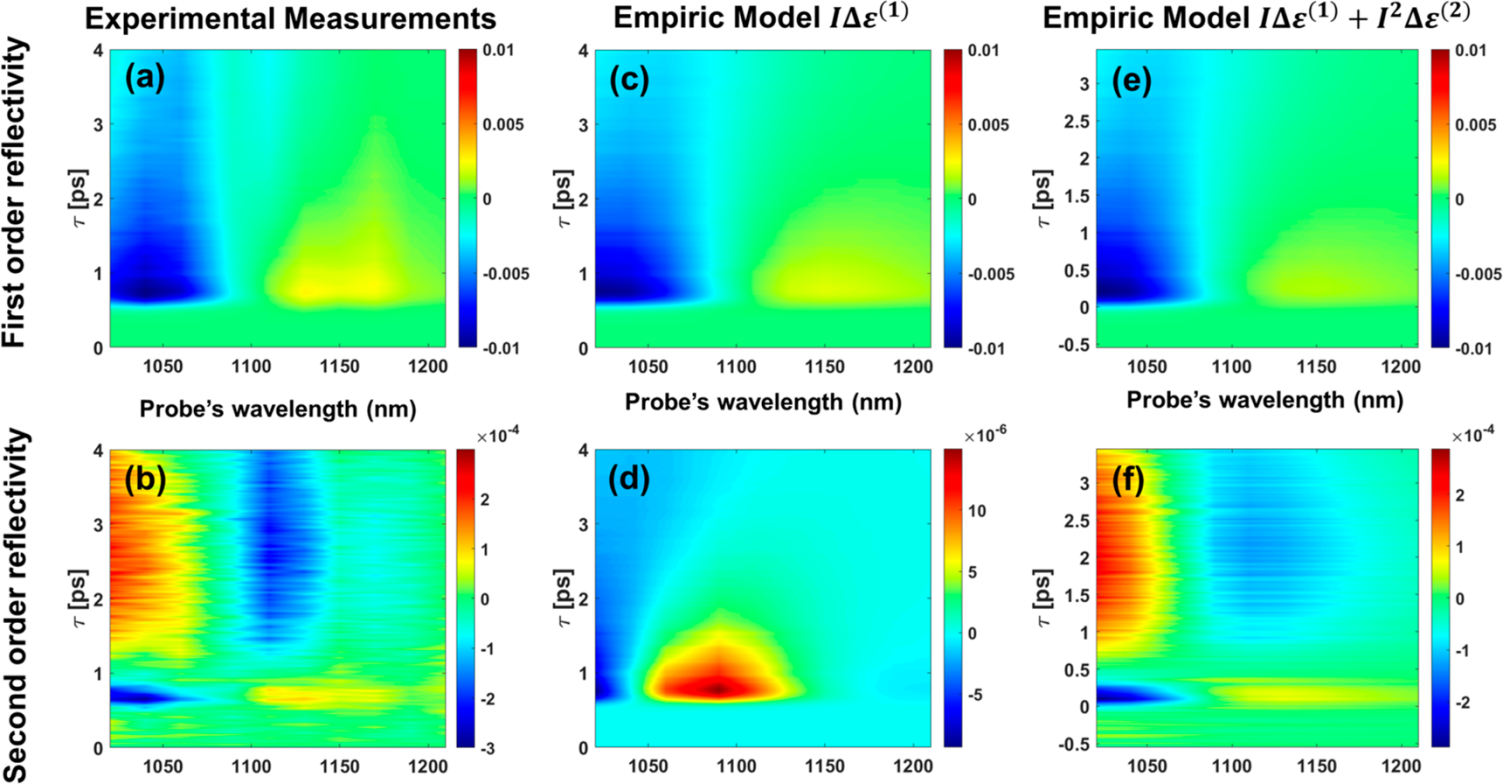
Experimental and modeled linear and nonlinear transient reflectivity maps from plasmonic nanostructures. Blue/red shades represent a reduction/increase in reflectivity due to pump excitation. (a, b) Experimental result of first- and second-order transient reflectivity measurements. (c, d) Transient reflectivity from an empirical model with a linear term only. This model could not fit the second-order transient reflectivity measurements. Note also that the second-order reflectivity changes are 2 orders of magnitude less than the experimental results. (e, f) Transient reflectivity model with second-order fitting terms. This allows a simultaneous fit to the first- and second-order transient reflectivity changes.

Under these assumptions, along with the use of a pure harmonic function modulated intensity, we run an optimization fit on **Δε**^(**1**)^ based on first-order reflectivity changes. We analyze the Fourier components of the Δ*R*/*R* response to extract the first- and second-order reflectivity changes, as shown in Figure 2(b,d). Although the fitted first-order differential reflectivity mimics the experimental results well, the resulting second-order reflectivity does not, establishing our claim that the measured second-order response cannot arise from dynamics that are only linear with the pump’s intensity.

We then extended our empirical model (eq 1) to include a quadratic dependence on the pump intensity, **Δε**^(**2**)^:
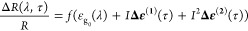
2Repeating the fitting process, considering the second-order dependence of **Δε**, with a pure harmonic function for the modulated intensity, we find an excellent simultaneous correlation for first- and second-order reflectivity changes, as depicted in Figure 2(e,f) (more details in Supporting Information SB).

We utilize the new experimental second-order response to better understand the physics and ultrafast dynamics of LSPR. Deciphering the complete transient response of an LSPR requires a comprehensive knowledge of the temporal dependence of the electronic distribution, which is affected by the interaction of the electrons with themselves and with the lattice’s phonons. These interactions, which depend on the electrons’ distribution in energy and momentum, are complex, and their modeling commonly requires numerical integration over time and space for the nanostructure.^24^ Simplified models have focused on the optical response of the electronic gas. For example, the basic two-temperature model (TTM), attributing dynamic temperature changes to electrons and the lattice, has been used to describe such systems.^25−27^ Here, we use the extended TTM model (eTTM)^13^ in which the population of the nonthermalized energy density (*N*) excited by the pump pulse and their thermalization lead to a rise in electron and lattice temperatures (*T*_e_ and *T*_l_, respectively). The transient dynamical changes of these parameters can then be used to calculate the change in the plasma frequency (ω_p_), the free electron collision rate (Γ), and the interband transition permittivity (ε_*∞*_), which offer a proper evaluation of the changes in the gold nanobar permittivity (ε_g_) and hence the change in the probe reflectivity.^28^

Initially, we fitted our experimental results using two versions of empirical kinetic eTTM models.^28,29^ In both cases, we get only a partial fit for the first-order reflectivity and a bad fit for the second-order reflectivity (Supporting Information SD). We conclude that in order to fit the full data set, the model for the evolution of the dielectric function induced by the dynamical processes should be modified. Our paradigm is that the physical system can be described faithfully only when simultaneous modeling of the first and second orders can be achieved over the full relevant time scale and probe-frequency scale (Supporting Information SB and SC).

To account for the changes in the reflectivity, we first extract the dielectric function of the LSPR in the nanobar prior to photoexcitation by using Mie scattering theory. We then modify the functional dependence of the plasma frequency and the decay with the transient parameters. The plasma frequency, ω_p_, which is proportional to the square root of the electronic density in Fermi energy, depends on both the electronic temperature (*T*_e_) and the lattice temperature (*T*_l_). Increases in *T*_l_ and *T*_e_ induce an expansion of the nanoparticles and thus reduce the electronic volume density.^9,29,30^ In our model, we also include the dependence of ω_p_ on the population of the nonthermalized electrons. Initially, we tried a linear fit (Δω_p_ ∝ *N*), with no success. By using the second term in a Taylor expansion (Δω_p_ ∝ *N*^2^), we were able to reproduce the second-order experimental results, with practically no effect on the first-order response. Accordingly, such an expansion was not needed in previous works that relied only on a first-order response (Supporting Information SD). Therefore, the plasma frequency is modeled as presented in eq 3
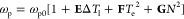
3where ω_p0_ is the plasma frequency at equilibrium and **E**, **F**, and **G** are fitting parameters. We note that although, theoretically, changes in plasma frequency due to the electronic temperature should be linear with *T*_e_,^28^ such fit parameters could not be found simultaneously for both the first- and second-order responses such that changes in plasma frequency will be proportional to *T*_e_ only (Supporting Information SE).

Our next step was to add a temperature dependence to the free-electron collision rate, Γ, which commonly includes electron–electron collisions, electron–phonon collisions, and electron–defect collisions.^10,29,30^ In addition to the constant term (Γ_0_), which considers electron-defect collisions and all other collisions, such as surface scattering, occurring independently of the temperature, we have added a term in which the electron–phonon collision rate depends linearly on the lattice temperature. Another term, proportional to the square of the electronic temperature, was added to account for electron–electron collisions. See eq 4,
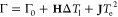
4where **H** and **J** are fitting parameters.

Finally, the presence of nonthermalized electrons has been shown to modulate interband transitions (d → s) to the conduction band by creating vacancies at energies close to the conduction band.^9^ As such, we have added an imaginary part to the dielectric function, which is proportional to *N* as shown in eq 5
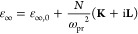
5where ε_*∞*,0_ is the dielectric function prior to pump excitation, **K** and **L** are fitting parameters, and ω_pr_ is the probe frequency.

Using the eTTM model, together with our empirical model, we successfully simulated both the first- and second-order Δ*R* as shown in Figure 3 (experiment, Figure 3(a,b); simulation, Figure 3(c,d)). The fit values and comparison to the existing literature can be found in Supporting Information Table S1. We emphasize that although our simulation faithfully describes the simultaneous temporal dependence and spectral dependence of the two sets of data, the individual fitting of each set provides a somewhat better fitting for the corresponding order (Supporting Information SF). A comprehensive comparison with existing theoretical values can be found in Supporting Information SC. Here, we focus on several key points. First, note that experimental results from linear Δ*R* correspond well with previous work on plasmonic structures.^9,11^ In addition, while the linear response shows a simple exponential relaxation of the change in Δ*R* over time, the nonlinear response shows a more complex behavior with Δ*R* changing its sign after a short ∼200–300 fs. We attribute these phenomena to the generation and depletion of the nonthermalized electron population.

**Figure 3:**
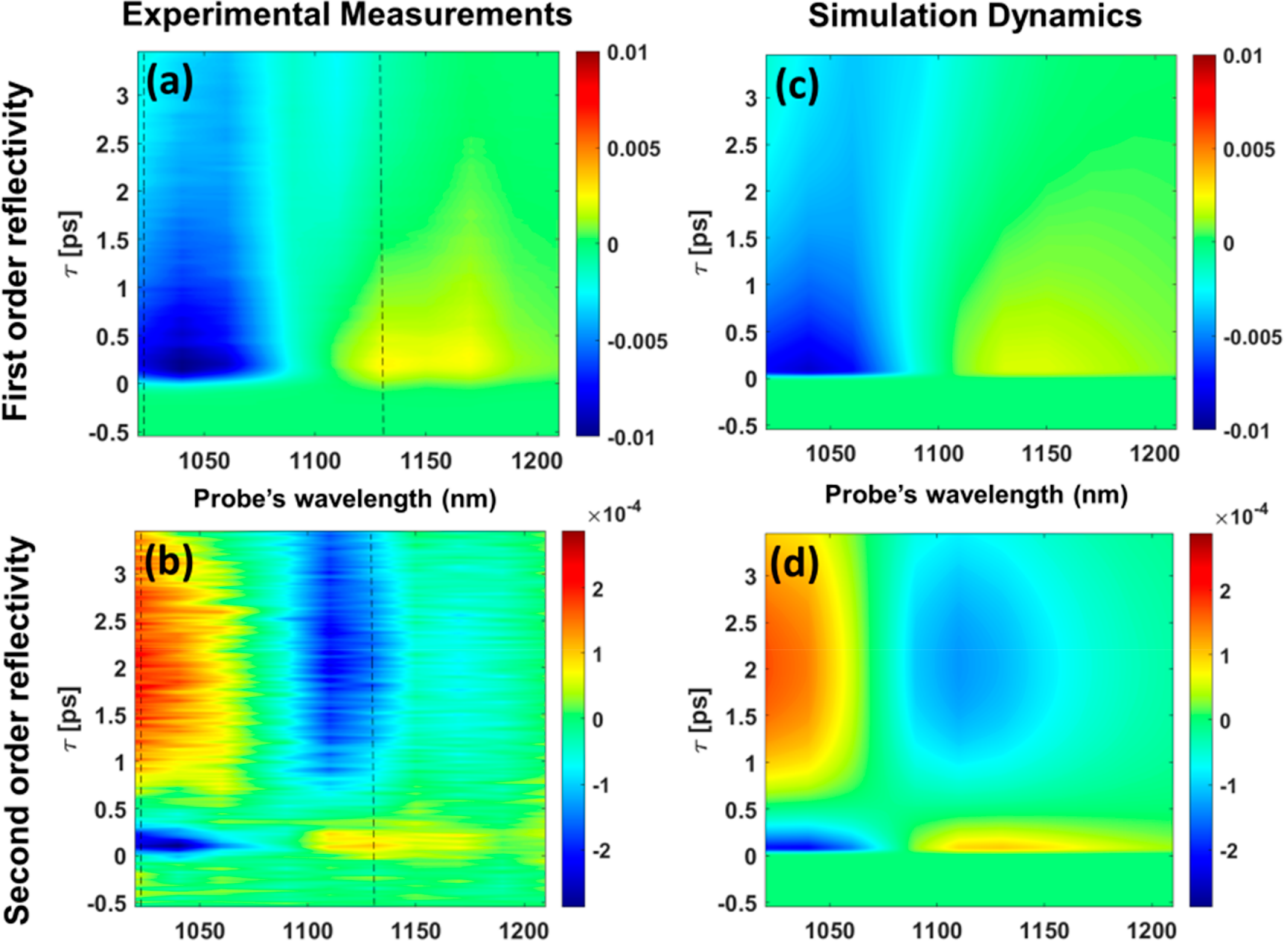
Transient reflectivity maps of normalized linear and nonlinear Δ*R* from experiment and the extended theoretical TTM model as a function of pump–probe delays and probe wavelengths. Blue/red shades represent a reduction/increase in reflectivity due to pump excitation. (a, b) Experimental results of linear and nonlinear Δ*R*. The dotted line marks the λ_probe_ cross-section shown in Figure 1c. (c, d) Result from the extended theoretical TTM model for linear and nonlinear Δ*R*.

Our model captures the dynamics observed from nonlinear changes in the real and imaginary parts of the dielectric function. In particular, the model captures the role of very hot electron and hole populations in the early stages (a few hundred fs) of the dynamics. Though a more rigorous approach of modifying the dielectric function has been presented by several groups,^9,11,28^ in this Letter we have decided to remain with the e-TTM framework. This allows us to focus on highlighting the added value of the high-order transient reflectivity information.

The functional dependencies of the optical constants are essential for the faithful modeling of the experimental results. Indeed, by removing any of them, we could not simultaneously fit both the first and second-order Δ*R* responses. The main difficulty was to recreate the fast 300 fs process after pump excitation in the second-order response (Figure 3b), where the Δ*R* response drops to 0 and changes its sign ∼500 fs after excitation. These time scales overlap with the fast creation and decay of nonthermalized electrons (*N*) and somewhat with the rapid rise in *T*_e_ due to pump interaction with the sample. This led us to extend the model by relating the imaginary part of ε_*∞*_ to *N* and as well as to ω_p_ and relating ω_p_ to *T*_e_^2^ (eq 3). We note that a model in which the plasma frequency changes linearly with *T*_e_ could not yield an adequate fitting (Supporting Information SE), emphasizing the importance of measuring the second-order response. The negative dependence of the plasma frequency on the square of the electronic temperature could arise from effects such as thermal expansion, which reduces the plasma frequency by decreasing the free carriers’ density.^30^

Finally, we qualitatively attribute the first- and second-order responses of the dielectric functions to the eTTM variables: *N*, *T*_l_, and *T*_e_. Figure 4a depicts the dynamics of these variables obtained with the optimization algorithm. Note that the energy is absorbed by the pump as nonthermalized energy (0–500 fs) and then transferred to the thermalized electronic gas (500–2500 fs) and finally transferred to the lattice (2500 fs). Assuming that the changes in the dielectric function have a relatively simple functional dependency on the eTTM variables, we can relate the changes in the real and imaginary parts of the first- and second-order dielectric functions to those variables.

**Figure 4:**
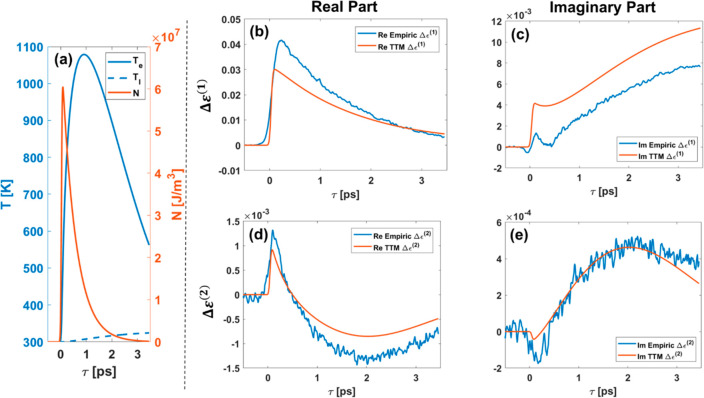
(a) Simulated eTTM dynamics of electron and lattice temperatures (blue and dashed blue curves, respectively) and nonthermalized electrons (orange curve) due to pump excitation. (b, c) First-order changes in real/imaginary gold permittivity, comparing values between an empiric and eTTM fit (blue and orange curves, respectively). (d, e) Second-order changes in real/imaginary gold permittivity, comparing values between empiric and eTTM fits (blue and red curves, respectively).

The first-order changes (real and imaginary) in the dielectric function, Δε^(1)^, are depicted in Figure 4(b,c). The fast rise in the real part can be attributed to the creation of nonthermal electrons, thus enabling the 5d → 6sp transition, as observed before.^11^ At longer times, where the electronic temperature and the lattice temperature rise, the real part decays to a smaller value, which can be attributed to thermal expansion. On the other hand, the imaginary part rises more slowly, indicating that this rise is not due to the electron–electron collision rate but is due to the increased electron–phonon collision rate.

The second-order change in the dielectric function shows an entirely different response. Surprisingly, the real part (Figure 4d) shows an abrupt increase in the first 500 fs, when then dynamics are dominated by the nonthermalized electrons. We suggest that this sharp increase is due to the transfer of free carriers to high energies, leading to a slight reduction in the Fermi-level electron–electron collision rate. After 300 fs, *Re*{**Δε**^(**2**)^} changes its sign to negative values. *Re*{**Δε**^(**2**)^} reaches its minimum after 2 ps, after which it decays back to zero at longer times. We attribute this decrease and rise to the smearing of the Fermi–Dirac distribution and a decrease in the plasma frequency.^30^

The second-order change in the imaginary part of the dielectric function (Figure 4e), *Im*{**Δε**^(**2**)^}, increases slowly and reaches its minimum value after 2 ps, after which it decays back to zero at longer times. We attribute these dynamics, dominant on the time scales of the rising electronic temperature, to electron–electron collision.^30^ We note that although this effect cannot be observed in the first-order response, because it is masked by first-order effects such as the 5d → 6sp interband transition, our approach allows its observation.

To conclude, we developed a unique pump–probe modality for the physical system’s linear and nonlinear ultrafast optical response. With a relatively mild extension of the P&P technique, we showed that the measurements of the second-order transient reflectivity response along with the conventional linear response can impose new insights in the interpretation of ultrafast dynamics in plasmonic nanostructures. Specifically, we show that in order to model the experimental results faithfully, it is mandatory to simultaneously fit the physical model over the full relevant time scale, frequency scale, and the first and second orders of the response. Specifically, for plasmonic nanobars, we found that the change in the dielectric function should include the transient changes in the electrons’ temperature *T*_e_, the plasma frequency ω_p_, and *N*. Our fitting relies on an empirical model. A profound detailed theory such as described in previous publications^9,11,28^ is desired. We believe that the method of high-order photoinduced reflectivity can go beyond the retrieval of the ultrafast nature of the photoinduced dynamics in plasmonic nanostructures. It can become a powerful tool in unraveling the transient ultrafast evolution and coupling in many other chemical and solid-state systems.

## Supporting Information Available

Additional information on experimental methods, modeling the LSPR of the gold nanobars, further detail on the extended TTM fitting process, effect of Δω_p_ ∝ *N*^2^ on first- and second-order responses, alternative fitting models, and the effects of different weighting of the linear and second-order data of the eTTM (PDF)
