# Supplementary material for: Second-Order Photoinduced Reflectivity for Retrieval of the Dynamics in Plasmonic Nanostructures
Source: Nano Lett. 2022 Jul 22;22(15):6179–85. doi: 10.1021/acs.nanolett.2c01478 (PMC9372992; doi:10.1021/acs.nanolett.2c01478)
Supplement: Supplementary file 1 — nl2c01478_si_001.pdf [file nl2c01478_si_001.pdf]

Supporting Information

Second-order Photo-induced Reflectivity for Retrieval of the Dynamics in Plasmonic Nano Structures

Dror Herhskovitz[†,‡], *Uri Arieli*[§,‡], *Sudarson Sekhar Sinha*[§,‡], *Ori Cheshnovsky*[*†,‡] *and Haim Suchowski*[*§,‡]

[†]Raymond and Beverly Sackler Faculty of Exact Sciences, School of Chemistry, Tel Aviv University 6997801, Israel.

[§]Raymond and Beverly Sackler Faculty of Exact Sciences, School of Physics & Astronomy, Tel Aviv University 6997801, Israel.

[‡]Tel-Aviv University center for Light-Matter-Interaction, Tel Aviv 6997801, Israel.

Table of Contents

SA. Methods

SB. Modeling properties of the localized surface plasmon resonance (LSPR) using Mie theory

SC. Extended Two-Temperature Model for calculating transient properties of the gold nano bars

SD. Effect of $\Delta\omega_p \propto N^2$ on First and Second Order Response

SE. Alternative Fitting Models

SF. The relative weights of first and second-order fitting

SA. Methods

For our experiment, we used a pulsed laser (Spectra-Physics Mai-Tai HP), with 100fs pulses, 80MHz rep-rate. 80% of the laser beam was then used to feed Oria OPO to generate the probe beam at variable wavelength, from 1010 to 1210 nm. The modulation of the pump beam was done via a commercial AOM (AA OPTO-ELECTRONIC MQ110-A3-UV, 90% diffraction efficiency) coupled with RF driver (MODA-110-B4-3660) fed by an Arbitrary Waveform generator AWG (Rigol DG4602). The beams were routed into a microscope setup using an infinity-corrected reflective objective (Thorlabs LMM-40X-P01, 0.5NA). Both pump and probe beams, at normal incidence to the nano bars, were linearly polarized along the long axis of the nano bars structures. A photodiode was used to measure the pump modulation and probe reflection (Thorlabs PDA36A at 0dB gain, with a bandwidth of 10MHz). The linearity of the photodetector was found to be better than 0.01% within the dynamic range of specific measurement, along the methodology described by Eppeldauer and coworker (1). Using a close loop PID process (2), the input function for the AOM was modified till the high harmonics content in the modulated pump signal was < 0.1%. We used a multi-demodulator lock-in amplifier to read and analyze the optical signal (Zurich Instrument UHFLI with 8 parallel demodulators).

To assure that the measured response is only due to pump modulation, we blocked the pump beam and verified the demodulated probe signal is equal to the instrument noise level when both beams are blocked. We have also regularly affirmed the "purity" of the pump modulation by the absence of second-order response on non-resonant surfaces (e.g. silver mirror and ITO cover slip) prior, during and after performing the measurement on the plasmonic nano bars.

SB. Modeling properties of the localized surface plasmon resonance (LSPR) using Mie theory

The first step in modeling the experimental results is to extract the dielectric function of the LSPR in the nano bar prior to photoexcitation using Mie scattering theory. To calculate those parameters, we used a parametric fit to determine the bars plasma frequency (ω_{p0}), electron inverse mean time between collisions (Γ_0), bar dimensions, host dielectric function (ε_h) and the ionic background contribution and short wavelengths interband transitions $\varepsilon_{\infty(0)}$, in room temperature, based on the measured reflection spectrum of the sample using eq. S1-S4.

$$\mathrm{L} = \int_0^{\infty} \frac{\mathbf{b_l} \cdot \mathbf{b_w} \cdot \mathbf{b_h}}{2(\mathrm{x} + \mathbf{b}_\mathbf{l}^2)^{\frac{3}{2}}(\mathrm{x} + \mathbf{b}_\mathbf{w}^2)^{\frac{1}{2}}(\mathrm{x} + \mathbf{b}_\mathbf{h}^2)^{\frac{1}{2}}} \mathrm{dx} \tag{1}$$

$$\varepsilon_{g0} = \boldsymbol{\varepsilon}_\infty - \frac{\omega_{p0}^2}{\omega_{pr}^2 + i\Gamma_0\omega_{pr}} \tag{2}$$

$$\alpha = \frac{\mathbf{b_l} \cdot \mathbf{b_w} \cdot \mathbf{b_h}\left(\varepsilon_{g0} - \boldsymbol{\varepsilon}_\mathbf{h}\right)}{\mathrm{L}\left(\varepsilon_{g0} - \boldsymbol{\varepsilon}_\mathbf{h}\right) + \boldsymbol{\varepsilon}_\mathbf{h}} \tag{3}$$

$$\sigma_0\left(\omega_{pr}\right) \propto \left(2\pi\omega_{pr}\right)^4 |\alpha|^2 \tag{4}$$

where L is depolarization factor, ε_{g0} is gold nano bar permittivity, ω_{pr} is the probe frequency, α are the bars polarizability, $\boldsymbol{b_l}, \boldsymbol{b_w}, \boldsymbol{b_h}$ are the bars length, width, and height, respectively, and σ_0 is the frequency dependent scattering cross section of the bars, prior to excitation.

In general, we assume a normalized pure harmonic function for the temporal pump intensity:

$$\mathrm{I(k)} = \frac{1}{2}\left[1 - \cos\left(\frac{2\pi \mathrm{k}}{\mathrm{m}}\right)\right] \tag{5}$$

where k is the index of the kth point in the pump modulation sequence, m is the total number of points in one modulation cycle.

In the linear model, for each pump-probe time delay, τ, and each point (k) in the modulation sequence, and each wavelength, ω_{pr}, we calculated the new scattering cross-section, $\sigma(k, \tau, \omega_{pr})$ using eq. S1-S4 and given that:

$$\varepsilon_g(\mathrm{k}, \tau, \omega_{\mathrm{pr}}) = \varepsilon_{g0} + \mathrm{I_k}\boldsymbol{\Delta\varepsilon^1} \tag{6}$$

where $\Delta\varepsilon^1$ is a complex fitting parameter denoting the linear change in ε_g due to the pump intensity.

We then assume that the overall reflectivity change, $\Delta R(k, \tau, \omega_{pr})$ is proportional to the difference in the scattering cross-section, $\Delta\sigma$, given by:

$$\Delta\mathrm{R}(\mathrm{k}, \tau, \omega_{\mathrm{pr}}) \propto \sigma(\mathrm{k}, \omega_{\mathrm{pr}}, \tau) - \sigma_0(\omega_{\mathrm{pr}}) \equiv \Delta\sigma \tag{7}$$

Taking eq. S1 and dividing left side of the equation by the maximum probe reflection prior to the pump pulse, and the right side by the maximum of σ_0, we get a unitless relation, and thus directly compare:

$$\frac{\Delta\mathrm{R}(\mathrm{k}, \tau, \omega_{\mathrm{pr}})}{\mathrm{R}} = \frac{[\sigma(\mathrm{k}, \omega_{\mathrm{pr}}, \tau) - \sigma_0(\omega_{\mathrm{pr}})]}{\sigma_0} \tag{8}$$

However, in our experiment, the photodiode response was measured in AC mode and therefore, our experimental data only consisted of ΔR. Based on other experiments, we scaled our data assuming a maximum ratio of $\frac{\Delta R}{R} = 1\%$,scaled the experimental results accordingly and could compare them with the simulated results.

Finally, to equate the model result to the measurement done by a lock-in amplifier, for each delay, τ, and probe frequency, ω_{pr}, we modulate the pump intensity as a pure harmonic function (eq. S%), and then use Fast-Fourier-Transform (FFT) to calculate in-phase and quadrature components

($X_n^{model}(\tau,\omega_{pr})$ and $Y_n^{model}(\tau,\omega_{pr})$ respectively), of $\frac{\Delta R}{R}$, as a function of τ and ω_{pr}, where n=1,2, represent different harmonics of the modulation, mimicking the operation of the lock-in amplifier. We can now compare the experimental results with those obtained from our model.

Since in our case, both experiment and simulation only give results in the in-phase component, we use X_n to calculate a figure of merit (FoM_n) for the model using eq. S9:

$$\mathrm{FoM_n} = \sum_{\tau,\omega_{pr}} \left|X_n^{model}(\tau,\omega_{pr}) - X_n^{exp}(\tau,\omega_{pr})\right|^2 \tag{9}$$

where $X_n^{exp}(\tau,\omega_{pr})$ and $X_n^{model}(\tau,\omega_{pr})$ are the experimental/model in-phase component of $\frac{\Delta R}{R}$ as a function of the nth demodulation harmonic, τ and ω_{pr}.

In the fitting process of the first model (eq. S6), for each τ, we iterate over $\Delta\varepsilon^1$ such as to minimize FoM_1.

For the non-linear empiric process, we repeat this procedure using eq. S10:

$$\varepsilon_g(k,\tau,\omega_{pr}) = \varepsilon_{g0} + I_k\Delta\varepsilon^{(1)} + I_k^2\Delta\varepsilon^{(2)} \tag{10}$$

where $\Delta\varepsilon^{(2)}$ is a complex fitting parameter denoting the second-order change in ε_g due to the pump intensity.

We then repeat the fitting process for $\Delta\varepsilon^1$ and $\Delta\varepsilon^2$, this time trying to minimize simultaneously both FoM_1 and FoM_2.

SC. Extended Two-Temperature Model for calculating transient properties of the gold nano bars

We use our extended Two-Temperature Model (eTTM) to simulate the population of non-thermalized electron (N), electron temperature (T_e) and lattice temperature (T_l) dynamics after pump pulse excitation, using a gaussian pump pulse (100fs FWHM). According to eq. S11, these calculations were repeated for different pulse intensities, simulating the modulation of the pump intensity as a pure harmonic function:

$$I_{k,\tau} = \mathbf{A}\cdot\frac{1}{2}\left(1-\cos\left(\frac{k}{2\pi m}\right)\right)\exp\left(\frac{\tau-\boldsymbol{\tau_0}}{0.33\mathrm{FWHM}}\right)^2 \tag{11}$$

where $\boldsymbol{A}$ is a fitting parameter for the pulse peak intensity, k is index of the kth point in the modulation sequence, m is the total number of points in the modulation cycle, τ is the delay between the pump and the probe, relative to pump peak time, $\boldsymbol{\tau_0}$ is a fitting parameter for pump pulse peak time and $FWHM$ is the full pump width at half maximum.

For each point (k) in the modulation sequence, the k-th pulse is used in eq. S12-S14 to calculate N, the density of the nonthermalized electron hole pairs, Te (the temperature of thermalized electrons) and T_l (the lattice temperature) as a function of τ.

$$\frac{dN}{d\tau} = -\mathbf{g_{ee}}N + \mathbf{P_{abs}}(k, \tau) \tag{12}$$

$$\frac{dT_e}{d\tau} = \mathbf{g_{ee}}N - (T_e - T_l)\mathbf{G_{eph}} \tag{13}$$

$$\frac{dT_l}{d\tau} = (T_e - T_l)\mathbf{G_{eph}} \tag{14}$$

Where $\boldsymbol{g_{ee}}$ is the electron heating rate by the non-thermalized electron-hole pairs and $\boldsymbol{G_{eph}}$ is the electron-phonon coupling constant and P_{abs} is the instantaneous absorbed power density. Once, N, T_e and T_l are known, we used those to calculate the time dependent $\omega_p(k,\tau)$, $\Gamma(k,\tau)$ and the $\varepsilon_\infty(k,\tau)$ according to eq. S15-S17.

$$\omega_p(k,\tau) = \omega_{p0}(1 + \mathbf{E}\Delta T_l + \mathbf{F}T_e^2 + \mathbf{G}N^2) \tag{15}$$

$$\Gamma(k,\tau) = \Gamma_0 + \mathbf{H}\Delta T_l + \mathbf{J}T_e^2 \tag{16}$$

$$\varepsilon_\infty(k,\tau) = \varepsilon_{\infty(0)} + \frac{N}{\omega_{pr}^2}(\mathbf{K} + i\mathbf{L}) \tag{17}$$

where ω_{p0}, Γ_0 and $\varepsilon_{\infty(0)}$ are the optical properties of the gold nano bars prior to pump excitation while $\boldsymbol{E}, \boldsymbol{F}, \boldsymbol{G}, \boldsymbol{H}, \boldsymbol{J}$, $\boldsymbol{K}$ and $\boldsymbol{L}$ are fitting parameters.

The modulated time-dependent ω_p, Γ and ε_∞ are then used again in eq. S1-S4 to calculate the modulated time-dependent scattering cross-section $\sigma(k,\tau,\omega_{pr})$ for a given point, k, in the modulation cycle. Finally, eq. S8 is used to calculate the modulated $\frac{\Delta R}{R}$ response.

To compare the model results to the experimental values, for each ω_{pr} and for each τ, we demodulate the resulting calculated $\frac{\Delta R}{R}$, as can be seen in Figure S1(a) for $\omega_{pr} = 1130\,nm$ and $\tau = 0.05\,ps$. Using Fast-Fourier-Transform (FFT) we can then extract the calculated in-phase and quadrature components ($X_n^{model}(\tau,\omega_{pr})$ and $Y_n^{model}(\tau,\omega_{pr})$ respectively), as a function of τ and ω_{pr}, where n=1,2 represent different harmonics of the modulating. Figure S1(b) shows the power spectrum ($|FFT|^2$) of the modulated σ.

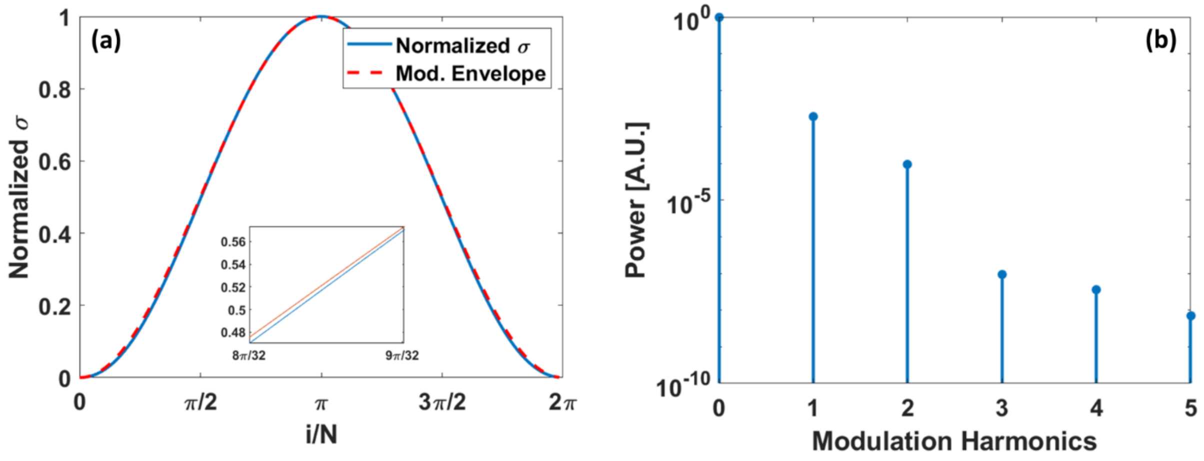

Figure S1 (a) Normalized scattering coefficient, σ, during one modulation cycle at $\tau = 0.05$ ps and $\omega_{pr} = 1130$ nm (blue line) and the pure harmonic modulation envelope of the pump pulses (dash orange line). Insert show zoom-in between $\frac{8\pi}{32} - \frac{9\pi}{32}$ *of the modulation cycle.* (b) The DC component + first five harmonics in the power spectrum of the modulated σ.

Since in our case, both experiment and simulation only give results in the in-phase component, we again use X_n to calculate a figure of merit (FoM_n) for the model using eq. *(18:(18)*

$$\mathrm{FoM_n} = \sum_{\tau,\omega_{pr}} \mathrm{a_n} \left| \mathrm{X_n^{exp}}(\tau, \omega_{pr}) - \mathrm{X_n^{model}}(\tau, \omega_{pr}) \right|^2 \tag{18}$$

where $X_n^{exp}(\tau, \omega_{pr})$ and $X_n^{model}(\tau, \omega_{pr})$ are the experimental/model in-phase component of the probe reflectance as a function of the nth demodulation harmonic, τ and ω_{pr}. a_n is a user input weight factor that enable us to favor specific harmonic over the other in the fitting process. In the extreme case of $a_1 = 1, a_2 = 0$, for example, the optimization process will try to fit the parameters based only on X_1^{exp}. This factor is added due to the different order of magnitudes in the values of the linear and non-linear data sets. In the case of using equal weights for both data sets ($a_n = 1, n = 1,2$) would have the fitting process mostly ignoring the error from the non-linear data set, X_2^{exp}, due to their much smaller values.

We repeat the fitting process iteratively to minimize the overall FoM for both the first and second harmonic signals, $\sum_1^n FoM_n$. The model result shown in our article (Figure 3(c-d)), were made with weights for data sets being, $a_1 = 1,\ a_2 = 35$, and an overall $FoM = 1.62 \times 10^{-3}$. The fit parameters are listed in Table S1.

Parameter	Fit Value	Literature Value	Units
P_{abs}	1.02e21	N/A	$Jm^{-3}s^{-1}$
τ_0	0.029	N/A	ps
g_{ee}	1.83e12	3.75e12 (3)	s^{-1}
G_{eph}	3.20e16	2.2e16 (3)	$J\,K^{-1}\,s^{-1}$
E	-6.82e-07	-2.11e-5 (4)	K^{-1}
F	-1.91e-10	N/A	K^{-2}
G	1.79e-20	N/A	m^6J^{-2}
H	2.71e+10	1.45e11 (4)	$s^{-1}K^{-1}$
J	9.14e+04	1.31e7 (4)	$s^{-1}K^{-2}$
K	1.48e+21	N/A	
L	1.75e+20	N/A	
ω_{p0}	8.97e+15	1.3e16 (5)	rad/s
Γ_0	6.88e+13	1.1e14 (5)	rad/s

Table S1 Value of physical parameter extracted using our eTTM simulation and comparison to literature values, where applicable.

Due to the additional parameters in our models, only the eTTM parameters g_{ee} and G_{eph} can be directly compared. As such, they show reasonable agreement with values in existing literature. As for the rest of the parameters, while we can't directly compare them, they do behave as one would expect. Namely, we that ω_p decreases with increased temperature (lattice and electrons) and increases with N. For Γ we see the opposite behavior, i.e. increases with the temperature.

SD. Effect of $\Delta\omega_p \propto N^2$ on First and Second Order Response

We have tried to fit the second-order response with only the linear dependence, yet with no success. This suggests that the second-order response should have a nonlinear relation with the time-dependent N. We have chosen to analyze this dependence by using the second term in a Taylor expansion, which can also fit other relations between N and the second-order response. For example, we could fit our data by introducing small nonlinearity in the dependence of the plasma frequencies on N (e.g., $N^{0.95}$). Such nonlinear relation probably stems due to the non-equilibrium dynamical evolution of the system in the first handstands fs, which should be further solved with

the ultrafast transient response of the Fermi-Dirac distribution toward electronic thermalization, a calculation that is beyond the scope of this work. Moreover, by adding the dependency of $\Delta\omega_p \propto N^2$ (eq. S5), we were able to recreate the fast transition in the second order response, while hardly affecting the first order response. For example, by running the model keeping all parameters fixed except parameter $= \mathbf{0}$, we see close to no change in the first order response, while no longer observing the fast sign change in the second order response (Figure S2).

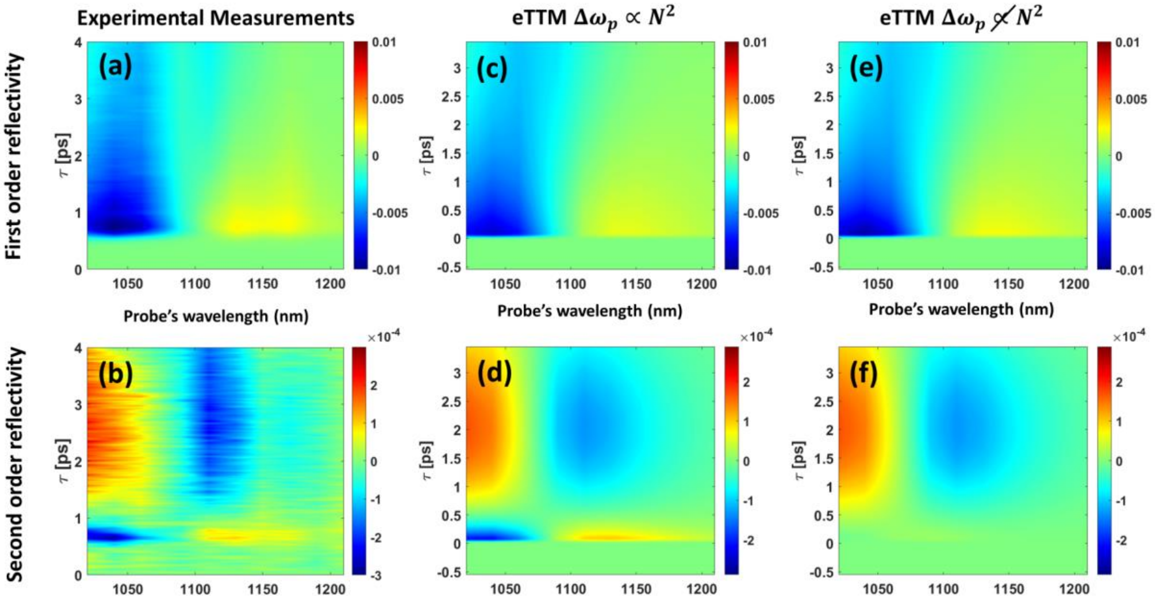

Figure S2 (a,b) Experimental results. (c,d) eTTM model using all fit parameters from Table S1. (e,f) eTTM model using parameter **G=0** while keeping all other parameters from Table S1, fixed.

SE. Alternative Fitting Models

As mentioned in our article, trying to fit the first order ΔR experimental results using existing eTTM models (4,6). However, we could not get a good agreement for the linear ΔR experimental results for probe wavelength higher than that of the LSPR resonance. As seen in Figure S3, while the models give good fitting for $\lambda_{probe} < 1100\ nm$, they fail on reproducing the positive ΔR

for $\lambda_{probe} > 1100\ nm$.

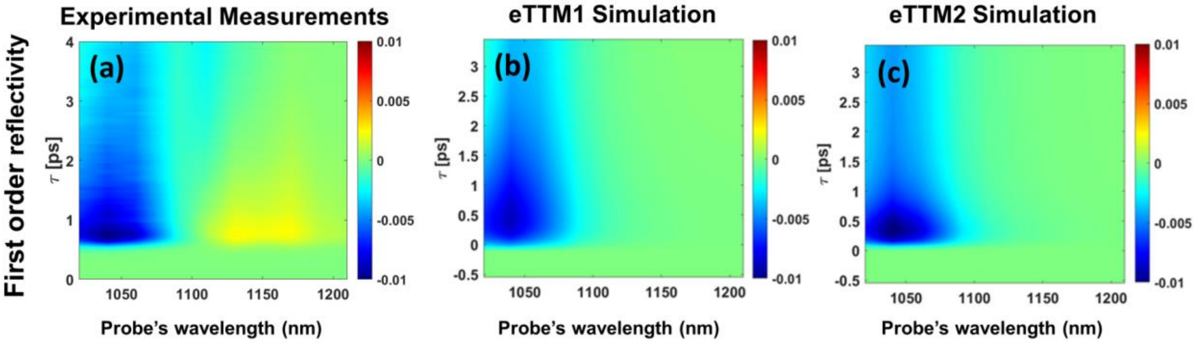

Figure S3 First order reflectivity from (a) our experimental result, (b) simulation using eTTM1 model taken from Ref. (4) and (c) eTTM2 model taken from Ref. (6).

We also tried to fit our eTTM with the change of equation S15 so that:

$$\omega_p(k,\tau) = \omega_{p0}\left(1 + \mathbf{E}N^2 + \mathbf{F}T_e + \mathbf{G}\Delta T_l\right) \tag{19}$$

i.e. the change of ω_p will depend linearly on T_e instead of T_e^2.

However, under these changes, while we manage to achieve almost as good fits with regard to the modulated $\frac{\Delta R}{R}$ (not shown), parameters **F** and **G** in Eq. S19 both converge to positive value (the dependence of ω_p on T_e and ΔT_l respectively) indicating the ω_p should increase with electron and lattice temperature, which is not physically sound.

SF. The relative weights of first and second-order fitting

Here we demonstrate the importance of the second order data set in fitting the transient $\Delta R/R$, and how the relative weight of the linear and nonlinear data sets in the optimization influences our fitting. As an example, we repeated the fitting process, fitting only with first-order data (a_1=1 a_2=0), only with the second-order data (a_1=0, a_2=1), and finally with a combination of both (a_1=1 a_2=35), as seen in Figure S4(a-h). While all results give a qualitative fit with the experimental results, the average relative errors (RE) for the different fittings were $RE_1 = 3.0\%, 18.3\%, 4.0\%$ for the **first order data sets** respectively, and $RE_2 = 18.3\%, 5.3\%, 5.7\%$ for the **second order data sets** respectively. Note that the 1/35 scale ratio balances the RE for both data sets.

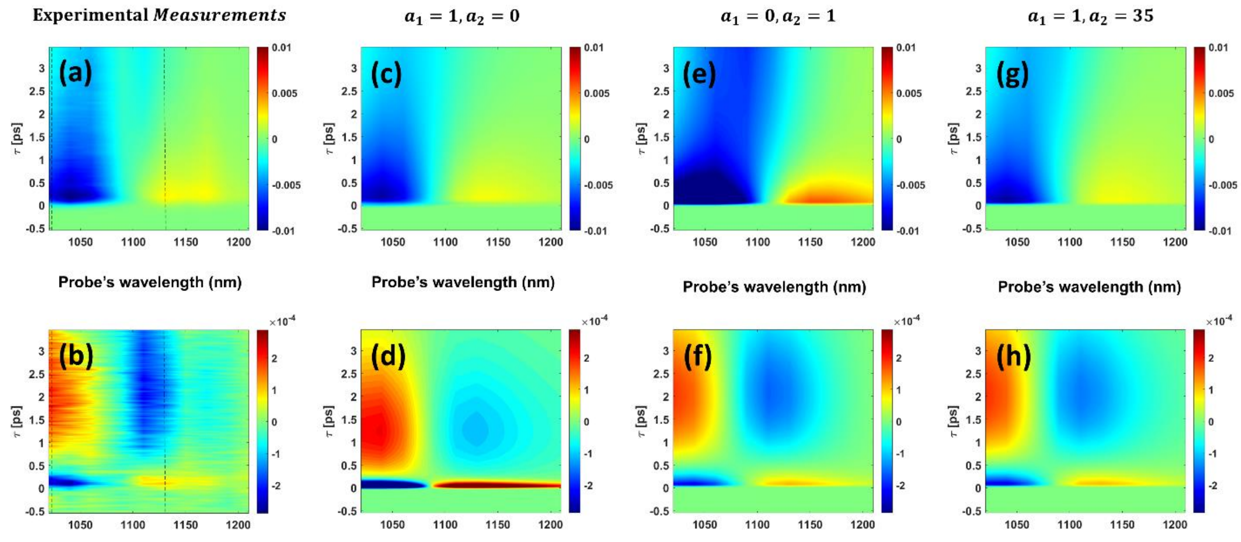

Figure S4 Fitting results from the extended TTM model for first order (top row) and second order (bottom row) $\Delta R/R$ using different weights for the first order and second order data in the optimization process. (a,b) The experimental results. (c,d) Model results when fitting the first order data only, (e,f) and when fitting the second order data only , respectively. (g,h) Model results when fitting both first and second order data with weight parameters providing comparable contributions to the errors of both data sets.

References

(1) Yoon, H. W.; Butler, J. J.; Larason, T. C.; Eppeldauer, G. P. Linearity of InGaAs Photodiodes. *Metrologia* **2003**, *40* (1 SPEC.), 1–5. https://doi.org/10.1088/0026-1394/40/1/003.

(2) Tzang, O.; Hershkovitz, D.; Nagler, A.; Cheshnovsky, O. Pure Sinusoidal Photo-Modulation Using an Acousto-Optic Modulator. *Rev. Sci. Instrum.* **2018**, *89* (12), 123102. https://doi.org/10.1063/1.5020796.

(3) Zavelani-Rossi, M.; Polli, D.; Kochtcheev, S.; Baudrion, A.-L.; Béal, J.; Kumar, V.; Molotokaite, E.; Marangoni, M.; Longhi, S.; Cerullo, G.; Adam, P.-M.; Valle, G. Della. Transient Optical Response of a Single Gold Nanoantenna: The Role of Plasmon Detuning. *ACS Photonics* **2015**, *2* (4), 521–529. https://doi.org/10.1021/PH5004175.

(4) Block, A.; Liebel, M.; Yu, R.; Spector, M.; Sivan, Y.; Abajo, F. J. G. de; Hulst, N. F. van. Tracking Ultrafast Hot-Electron Diffusion in Space and Time by Ultrafast Thermomodulation Microscopy. *Sci. Adv.* **2019**, *5* (5), eaav8965. https://doi.org/10.1126/SCIADV.AAV8965.

(5) Cai, W.; Shalaev, V. *Optical Metamaterials: Fundamentals and Applications*; Springer New York, 2010. https://doi.org/10.1007/978-1-4419-1151-3.

(6) Schirato, A.; Maiuri, M.; Toma, A.; Fugattini, S.; Zaccaria, R. P.; Laporta, P.; Nordlander, P.; Cerullo, G.; Alabastri, A.; Valle, G. Della. Transient Optical Symmetry Breaking for

Ultrafast Broadband Dichroism in Plasmonic Metasurfaces. *Nat. Photonics 2020 1412* **2020**, *14* (12), 723–727. https://doi.org/10.1038/s41566-020-00702-w.
